# MMP-2 and MMP-9 plasma levels are potential biomarkers for indeterminate and cardiac clinical forms progression in chronic Chagas disease

**DOI:** 10.1038/s41598-019-50791-z

**Published:** 2019-10-02

**Authors:** Nayara I. Medeiros, Juliana A. S. Gomes, Jacqueline A. Fiuza, Giovane R. Sousa, Eliane F. Almeida, Renata O. Novaes, Virgínia L. S. Rocha, Ana T. Chaves, Walderez O. Dutra, Manoel O. C. Rocha, Rodrigo Correa-Oliveira

**Affiliations:** 1 Imunologia Celular e Molecular, Instituto René Rachou, Fundação Oswaldo Cruz, Belo Horizonte, MG Brazil; 2 Laboratório de Biologia das Interações Celulares, Departamento de Morfologia, Universidade Federal de Minas Gerais, Belo Horizonte, MG Brazil; 3 Programa de Pós-Graduação em Infectologia e Medicina Tropical, Faculdade de Medicina, Universidade Federal de Minas Gerais, Belo Horizonte, MG Brazil; 4 Instituto Nacional de Ciência e Tecnologia em Doenças Tropicais - INCT-DT, Belo Horizonte, Minas Gerais Brazil

**Keywords:** Parasitic infection, Parasitic infection, Parasite host response, Parasite host response, Predictive markers

## Abstract

One of the major challenges in chronic Chagas disease is to understand the mechanisms that predict the clinical evolution from asymptomatic to severe cardiac clinical forms. Our cohort consisted of twenty-eight Chagas disease patients followed for twenty years. Plasma levels of MMP-2 and MMP-9 gelatinases and TIMPs were evaluated by multiplexed immunoassay at two points in time with an average interval of six years. MMP-2 plasma levels, but not MMP-9, increased in cardiac patients over time. TIMP-1 levels diminished in cardiac patients, while TIMP-3 dropped in asymptomatic patients in the course of the evaluated interval. An inversion of time lines was observed relative to the clinical asymptomatic and cardiac forms for MMP-2. Receiver Operating Characteristic (ROC) curve analysis identified MMP-2 as a biomarker to distinguish asymptomatic from cardiac clinical forms, while MMP-9 is a biomarker that segregates infected from non-infected patients. We have pointed out that MMP-2 and MMP-9 together can predict clinical evolution in Chagas disease. MMP-2 was suggested as a biomarker for fibrosis replacement in early remodeling and a sensitive predictor for initial changes in asymptomatic patients that may evolve into the cardiac clinical form. MMP-9 seems to be a biomarker for late fibrosis and severe cardiac remodeling in cardiac patients.

## Introduction

Chagas disease, or American trypanosomiasis, is a tropical parasitic disease caused by the protozoan *Trypanosoma cruzi*^1^. Once totally confined to the Region of the Americas, Chagas disease has spread to other continents over the last century mainly as a result of enhanced travel and global population movement to and from Latin America^2^.

Human Chagas disease shows a variable clinical presentation. The acute phase is characterized by high parasitemia, which is easily detected by direct blood examination^3^. The chronic phase, generally starting with a lengthy, asymptomatic, or latent clinical form, is called the indeterminate form (IND)^4,5^. Whereas most *T*. *cruzi* infected patients persist in IND indefinitely, about 40% of them may develop lesions in different organs 10–30 years after initial infection, principally in the heart, the cardiac form (CARD) of the disease^6,7^.

One of the major challenges in chronic Chagas disease is to identify a mechanism of intervention that is capable of predicting the clinical evolution of IND to CARD or minimizing the effects of fibrosis developing in the heart. Therefore, significant effort is given to the identification of molecular biomarkers that can be used to predict the evolution of the disease and make a clinical prognosis.

Our group has previously described that matrix metalloproteinases (MMPs) and tissue inhibitors of MMPs (TIMPs) are potential biomarkers of chronic changes in Chagas heart disease^8–10^. MMPs are a group of zinc-dependent endopeptidases that control extracellular matrix (ECM) synthesis and degradation by cleavage of collagen, laminin, and other molecules^11^. An imbalance between MMPs and TIMPs has been shown to be involved in some diseases, such as cancer^12^, arthritis^13^, and obesity^14^. MMPs also participate in the inflammatory process due to their proteolytic activity in cytokine activation^15^.

MMP-2 and MMP-9 gelatinases are the primary MMPs described in heart remodeling by distinct triggers^16,17^. It has been proposed that gelatinases may have an opposite effect on inflammation/regulation and cardiac remodeling in Chagas disease^10^, as has been suggested in other diseases^18^. We previously proposed that MMP-2 participated in such regulation, offering a protective role for cardiac damage in IND patients, and would be a good marker for the onset of changes in the heart^9^. Conversely, MMP-9 could be used as a marker for more severe changes in the heart and would be associated with inflammation and fibrosis^8^.

In this study, we evaluate the plasma levels of MMP-2 and -9 gelatinases and TIMPs in a cohort of patients with chronic Chagas disease. These molecules are analyzed for the first time during their progression in the clinical forms, a fundamental step that will allow their assessment as putative prognostic biomarkers of chronic Chagas disease.

## Results

### MMP-2 levels decrease in cardiac patients

**Figure 1:**
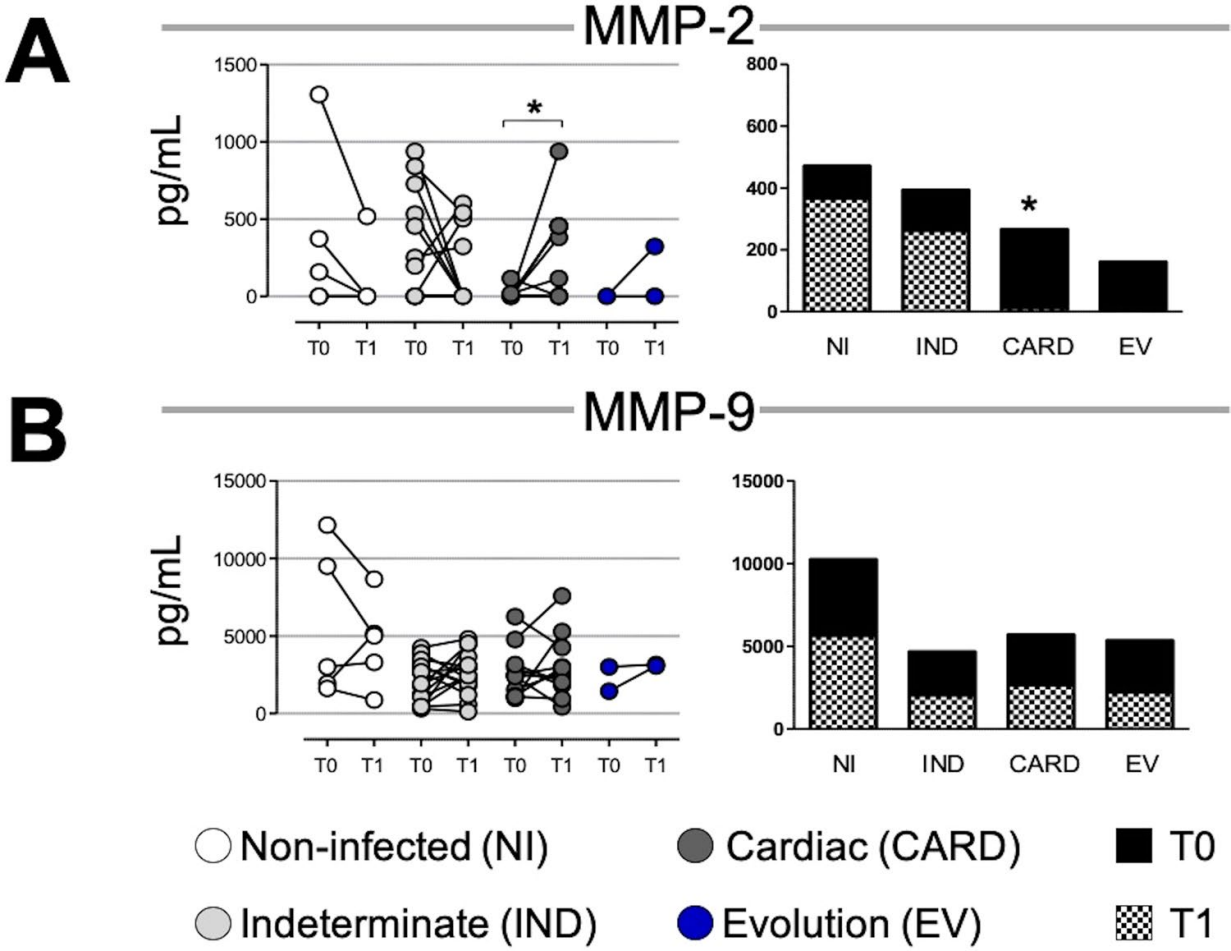
Matrix metalloproteinases (MMP)-2 and MMP-9 plasma levels over time. Paired points graphs demonstrated concentration (pg/mL) of MMP-2 (**A**) and MMP-9 (**B**) in plasma from non-infected individuals (NI, white, n = 5), patients with indeterminate (IND, gray, n = 15) and cardiac (CARD, dark gray, n = 11) clinical forms of Chagas disease, and from patients that had evolution of clinical forms during the cohort (EV, blue, n = 2). Proportion graphs demonstrated median for time zero (T0, black) over median for time one (T1, checkered). Statistical differences (p ≤ 0.05) between times were obtained by Wilcoxon signed-rank test and Mann-Whitney test, showed by asterisk (*).

Our data show that MMP-2 plasma levels increase significantly in T1 as compared to T0 in the CARD group (Fig. 1A). However, when we evaluated the proportion between times, we observed that NI and IND showed a higher proportion of MMP-2 in T1, while the CARD and EV groups showed a higher proportion in T0 (Fig. 1A).

### MMP-9 levels appear to be unchanged over time

We demonstrated there were no differences in MMP-9 plasma levels between times in the NI, IND, and CARD groups (Fig. 1B). Likewise, no differences were observed in the proportion of MMP-9 plasma concentration between times in any of the groups (Fig. 1B).

### TIMPs are differentially abundant in chronic chagas disease

**Figure 2:**
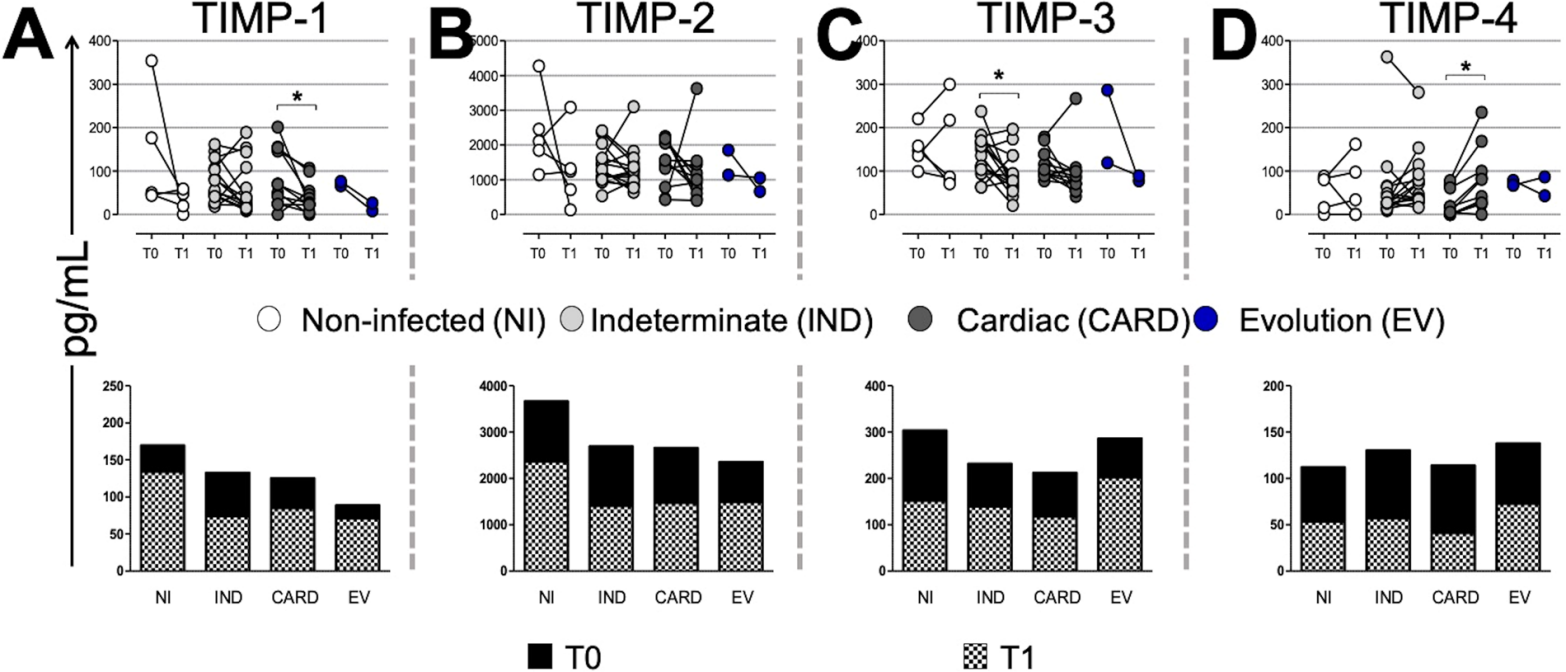
Tissue inhibitor of metalloproteinases (TIMP) plasma levels over time. Paired points graphs demonstrated concentration (pg/mL) of TIMP-1 (**A**) TIMP-2 (**B**) TIMP-3©, and TIMP-4 (D) in plasma from non-infected individuals (NI, white, n = 5), patients with indeterminate (IND, gray, n = 15) and cardiac (CARD, dark gray, n = 11) clinical forms of Chagas disease, and from patients that had evolution of clinical forms during the cohort (EV, blue, n = 2). Proportion graphs demonstrated median for time zero (T0, black) over median for time one (T1, checkered). Statistical differences (p ≤ 0.05) between times were obtained by Wilcoxon signed-rank test and Mann-Whitney test, showed by asterisk (*).

Our results showed that TIMP-1 levels were decreased in T1 as compared to T0 in the CARD group (Fig. 2A). No differences in the TIMP-1 proportion were observed between the groups in T1 and T0 (Fig. 2A).

No statistical differences were shown in TIMP-2 levels between times in all groups or in the proportion evaluation (Fig. 2B).

TIMP-3 plasma concentration significantly decreased in T1 as compared to T0 in the IND group, but the proportion graph did not show any statistical differences between time points (Fig. 2C).

We also demonstrated a significant increase of TIMP-4 levels in T1 as compared to T0 in the CARD group, while no statistically significant differences were seen in the proportion between the time points (Fig. 2D).

### MMP-2 high producers curve showed an overturn of time lines in IND and CARD

**Figure 3:**
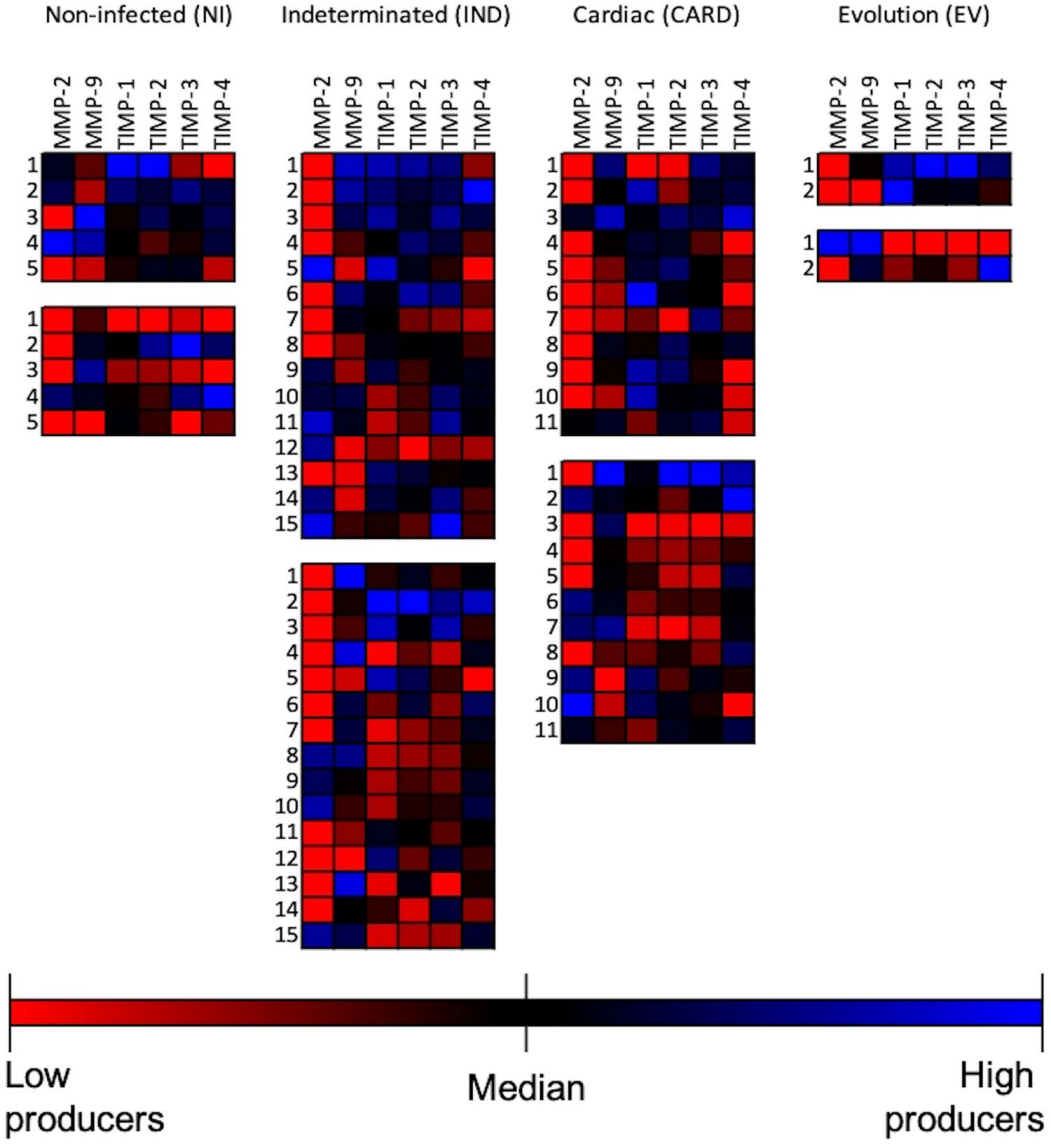
Low and high producers for Matrix metalloproteinases (MMP) and Tissue inhibitor of metalloproteinases (TIMP). Heatmaps demonstrated low (red), median (black), and high (blues) producers for MMP-2, -9, TIMP-1, -2, -3 and -4. Groups were separated into non-infected individuals (NI, n = 5), patients with indeterminate (IND, n = 15) and cardiac (CARD, n = 11) clinical forms of Chagas disease, and from patients that had evolution of clinical forms during the cohort (EV, n = 2). For each group, two boxes are demonstrated: upper box indicates time zero (T0) and lower box indicates time one (T1). The numbers identify the patient.

We used heatmaps to differentiate high and low producers using the median values. Our data clearly show the segregation of different profiles of low and high producers in all evaluated groups (Fig. 3). We observed a predominance of high producers for all molecules (in blue) in T0 as compared with the majority of low producers (in red) in T1 in all groups (Fig. 3). This difference was evident in the EV group when we evaluated the color contrast between T0 and T1 boxes (Fig. 3).

**Figure 4:**
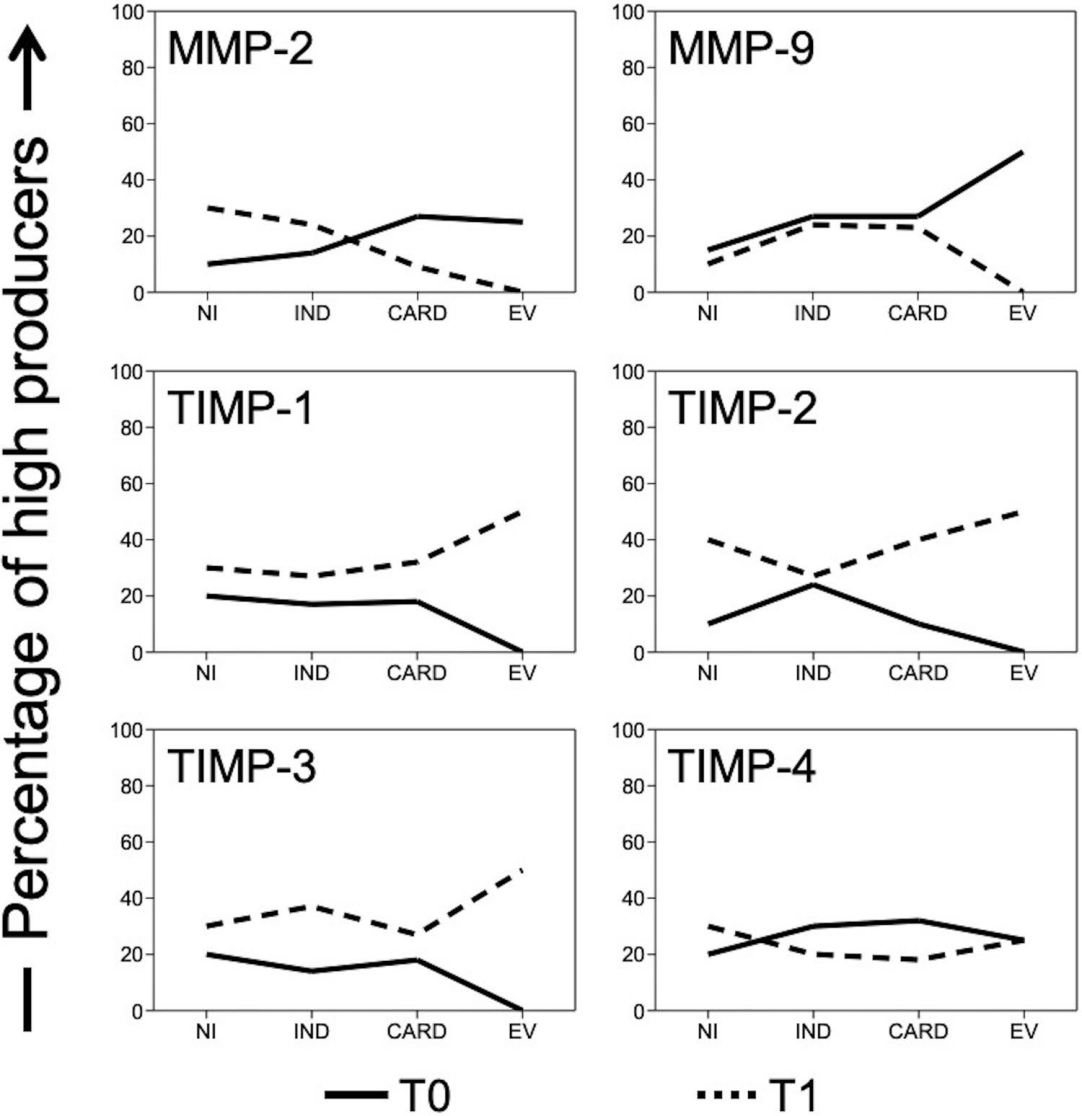
Percentage of high producers. Calculated percentage of high producers for non-infected individuals (NI, n = 5), patients with indeterminate (IND, n = 15) and cardiac (CARD, n = 11) clinical forms of Chagas disease, and from patients that had evolution of clinical forms during the cohort (EV, n = 2) was represented as a curve graph. The solid line showed the percentage of high producers in time zero (T0) and the dotted line the time one (T1) per group.

We measured the high producers by calculating their percentile as shown in the curves in Fig. 4. The percentage of MMP-9 and TIMP-4 high producers was higher in T0 as compared to T1 in all groups (Fig. 4). The percentage of TIMP-1, -2, and -3 high producers was higher in T1 than in T0 for all groups (Fig. 4). Only in the MMP-2 curve was an inversion of the time lines in IND and CARD clinical forms observed (Fig. 4). The percentage of MMP-2 high producers in IND patients was higher in T1 than in T0, while CARD patients were higher in T0 than in T1 (Fig. 4).

### MMP-2 is an important biomarker for cardiac evolution in chronic chagas disease

**Figure 5:**
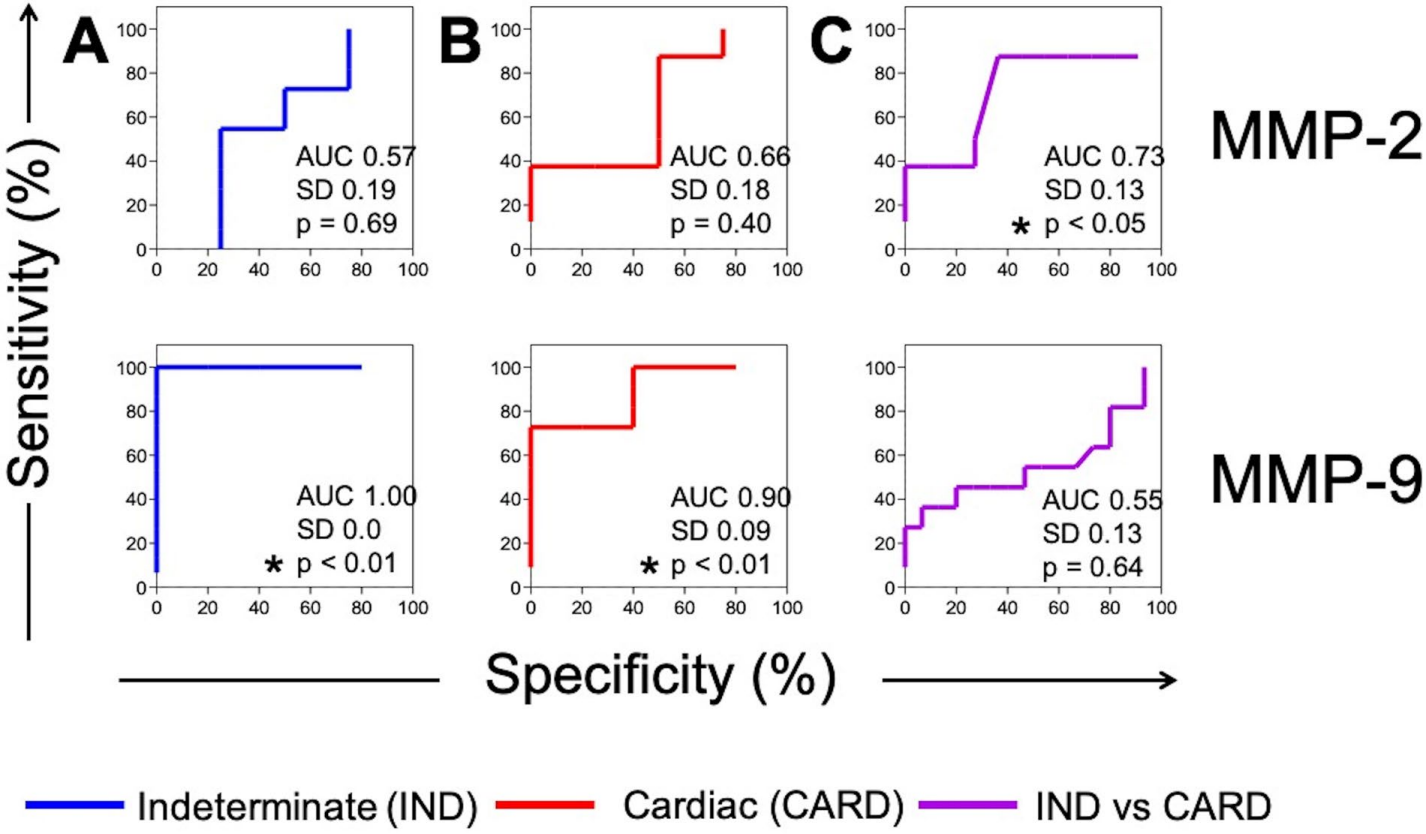
Biomarker performance measurement. Receiver Operating Characteristics (ROC) curve analysis was calculated to determine the potential for plasma MMP-2 and MMP-9 levels to segregate: (**A**) non-infected individuals (NI) from patients with indeterminate (IND) clinical form; (**B**) NI from patients with cardiac (CARD) clinical form; and (**C**) IND from CARD patients. All percentage values to sensitivity and specificity were shown. The measure of separability was demonstrated by the area under the curve (AUC) with 95% confidence interval. Statistical significance (p ≤ 0.05) was showed by asterisk (*).

We used the Receiver Operating Characteristic (ROC) curve to evaluate the potential of MMP-2 and MMP-9 as putative biomarkers for the evolution of the clinical forms. Our data show that MMP-2 does not have enough sensitivity and specificity to segregate infected groups (IND and CARD) from the NI group (Fig. 5A,B). Nevertheless, differences in MMP-2 levels were statistically significant and able to isolate IND from CARD patients with a high degree of sensitivity and specificity (Fig. 5C).

### MMP-9 as a biomarker of cardiac morbidity in chronic chagas disease

Our data also demonstrated that MMP-9 segregates chronic infected patients from the NI group with significant sensitivity and specificity, regardless of their clinical forms (Fig. 5A,B). Nevertheless, significant differences that could categorize IND from CARD were not observed in the ROC curve (Fig. 5C).

## Discussion

*T*. *cruzi* infection is usually followed by a long and clinically silent period before the development of an overt clinical presentation of Chagas lesions and severe heart disease. Characterizing biomarkers that indicate the transition between clinical forms and cardiomyopathy progression may be used to address the clinical procedures aimed at minimizing cardiac changes and their associated risks, as well as the use of therapeutic medication. Here, we characterize the MMP-2 and MMP-9 gelatinases as putative biomarkers of the evolution of clinical forms in Chagas disease.

Increased synthesis of MMPs comes from an imbalance between TIMP levels, resulting in an increase of collagen deposition, ECM cross-linking, and fibrosis. Fibrosis impairs contractility and disrupts the chemoelectrical conduction in the heart, leading to arrhythmias, local microfibrillations, and inefficient contraction^19,20^. Changes in the ECM may be associated with ventricular dysfunction and structural changes observed in both human and experimental models of *T*. *cruzi*-induced cardiomyopathy^8,9,21–26^.

Bocchi *et al*. emphasized the importance of biomarkers being specific for *T*. *cruzi* infection or its validation to Chagas heart disease as compared with other cardiomyopathies^27^. We agree that some biomarkers, including MMP-2 and MMP-9, may reflect myocardial injury or heart failure regardless of *T*. *cruzi* infection. Nevertheless, we point out, based in our^8,9^ and other^22,26,28^ previous studies, that gelatinases play a pivotal role in chronic Chagas disease when associated with the dichotomic immune response observed in infected patients. MMP-2 and MMP-9 gelatinases were observed during experimental *T*. *cruzi* infection, and MMP blockage leads to a reduction in myocardial inflammation and patients’ survival rate during the acute phase of infection^21^. In human Chagas disease, MMP-2 has been previously associated with regulatory responses in IND patients by correlations with regulatory cytokines, especially IL-10, whereas MMP-9 has been associated with inflammatory response and cardiomyopathy^8,9^.

MMP-2 can regulate remodeling after myocardial infarction^29^ and chronic pressure overload^30^, when remodeling improves without inflammation. Decreased MMP-2 activity contributed to a rise in myocardial fibrosis in a cardiomyopathy animal model for diabetes^31^. Hardy and colleagues described when MMP-2 activity falls below the baseline, there is an increase in pro-inflammatory cytokine production and cardiac secretion of phospholipase A2, which results in systemic inflammation and downstream dysregulation^32^.

Our data showed different plasma levels only for MMP-2 in CARD groups at the evaluated time points. Curiously, patients with higher levels of MMP-2 in T0 showed a decrease in T1, while patients with lower MMP-2 levels in T0 showed a high level in T1. Our data suggest that, in the different time points and patient groupings, there is an important difference in the median that may be indicative of the evolution of heart disease. The mean time proportions showed an increase in the proportion of MMP-2 over time in the NI and IND groups, while in the CARD and EV groups, the MMP-2 proportion was higher in the first observation. This finding does not characterize an evolution, given that CARD patients already have severe heart disease and the EV groups are too small to support making this statement, but it shows that CARD patients lose MMP-2 balance as compared to IND patients between times.

We believe that MMP-2 represents a transition period between clinical forms and that changes in its production may signal initial changes and remodeling of cardiac disease. Although MMP-2 and -9 may lead to these changes, they have different interactions with several ECM macromolecules, suggesting they may be involved in different stages of remodeling. MMP-2, digests fibronectin, laminin, and type III collagen^33^, while the α2 chains of type I collagen are degraded only by MMP-9^34^.

It has been proposed that the fibrotic response in heart remodeling may be classified into two types, namely replacement and reactive fibrosis. Replacement fibrosis is characterized by early scar formation, an important process in preventing ventricular wall rupture after cardiac damage^35^, in which collagen III levels were increased for early remodeling^36^. The increased mechanical stress after cardiac injury also induces connective tissue expansion in areas remote from the damage. Reactive fibrosis in the uninjured myocardium leads to altered chamber compliance and increased ventricular stiffness, thereby compromising cardiac output^35^ by the late construction of strong fibers with type I collagen^36^.

We showed that the NI and IND groups have an elevated percentage of patients that are high MMP-2 producers in T1 as compared to CARD, which presented the greatest percentage of MMP-2 high producers at the T0 time point. Considering the specificity for type III collagen and the contribution of MMP-2 to replacement fibrosis in the early remodeling, we reiterate that MMP-2 may possibly be an important biomarker for the initial lesions at the moment of clinical transition. This is further supported by the observation that MMP-2 segregates IND from CARD patients with high sensitivity and specificity in the ROC curve.

Conversely, MMP-9 is related to inflammation and fibrosis, mainly through the synthesis and deposition of type I collagen in reactive fibrosis^35^. MMP-9 levels are increased in a number of cardiovascular diseases, including hypertension, atherosclerosis, and myocardial infarction^37,38^. Some authors have focused on the importance of MMP-9 as damage and remodeling biomarkers in cancer, multiple sclerosis, epilepsy, and heart disease^38^. Matsumoto *et al*. showed that MMP-9, but not MMP-2, is involved in cardiac remodeling during the development and progression of auto-immune myocarditis and subsequent dilated cardiomyopathy^38^.

We propose MMP-9 as a potential biomarker of late fibrosis, where cardiac remodeling results in heart failure, apical aneurysm of the left ventricle, and hypertrophy. Indeed, MMP-9 segregated infected patients from the NI group with high sensitivity and specificity in the ROC curve, yet no differences were detected in plasmatic concentration in the groups over time. We suggest that MMP-9 activity could be controlled by TIMP-1 levels detected over time in the NI and IND groups. However, the CARD group showed a decreased level of TIMP-1 in T1 as compared to T0, which may favor MMP-9 activity in late cardiac remodeling, since TIMP-1 has a higher affinity for MMP-9 blockade.

Four TIMPs inhibit all MMPs, but they have some selectivity. In addition to TIMP-1 with MMP-9 affinity, TIMP-2 and TIMP-4 preferentially block MMP-2, but TIMP-3 has no reported MMP specificity^39^. The decrease of TIMP-3 levels in the IND group over time may have no relation to the gelatinases, but rather with other enzymes. TIMP-4 has its greatest *in situ* effect, especially on MMPs in the heart^39^, suggesting a response to cardiac damage and MMP-2 inhibition in the CARD group by a significant increase over time. Nevertheless, TIMPs do not seem to have a potential to be used as biomarkers in a low/high producer analysis with the data from the ROC curve.

In conclusion, our results point out that MMP-2 and MMP-9 together could be a tool for predicting clinical evolution in Chagas disease. Contrary to our previous suggestion^8,9^, MMP-2 does not appear to be a protective factor in IND patients. We believe that MMP-2 could be a potential biomarker for replacement fibrosis in early remodeling and a sensitive predictor for initial alterations in IND patients that may evolve into the cardiac clinical form. MMP-9 seems to be a potential biomarker for late fibrosis and severe cardiac remodeling in CARD patients. We propose that doses of gelatinases in clinical management, coupled with evaluations of the physiology of the heart, may be an important prognosis predictor and facilitate preventive measures in patients with Chagas disease.

## Methods

### Study population

This is a cross-sectional retrospective cohort study in which longitudinal samples from 28 patients with chronic Chagas disease were analyzed. The study population comprised patients from endemic areas within the state of Minas Gerais, Brazil, who were referred to the Referral Outpatient Center for Chagas Disease at the Clinical Hospital of the *Universidade Federal de Minas Gerais* (UFMG), Belo Horizonte, Minas Gerais, Brazil. Plasma samples were obtained from patients with Chagas disease between May 1994 and September 2014. The diagnosis of Chagas disease required at least two positive serologic tests for antibodies against *T*. *cruzi* (indirect hemagglutination, indirect immunofluorescence, or enzyme-linked immunosorbent assay). All patients underwent a thorough clinical evaluation at several time points during the study.

Inclusion criteria included the diagnosis of IND defined as asymptomatic individuals, in sinus rhythm, with no significant changes in electrocardiography, chest radiograph and echocardiogram, and diagnosis of CARD characterized by the echocardiographic findings of a dilated left ventricle with impaired ventricular systolic function. LVEF, LVDD, and E/e′ ratio were used as parameters of the left ventricular function, where LVEF <55% and LVDD/body surface area ≥31 mm were used to define CARD patients^40^. Individuals with any other chronic inflammatory disease, thyroid dysfunction, valvular heart disease, coronary artery disease, systemic arterial hypertension, chronic obstructive pulmonary disease, hydroeletrolytic disorders, renal insufficiency, diabetes mellitus, alcoholism, and other infectious diseases were excluded from the study.

Five healthy individuals from a non-endemic area for the disease, matched to infected patients by age and sex, with negative serological tests for the infection, were included as a non-infected (NI) group. None of the patients were undergoing etiological treatment nor had any of them been previously treated for *T*. *cruzi* infection.

The population, evaluation interval between collects, time zero (T0), and time one (T1) are shown in Table 1. Two infected patients had evolution of clinical forms during the cohort study to the CARD form. Although it is not possible to perform statistical analysis using such a small group, we grouped the patients into evolution (EV) groups for a qualitative evaluation.

**Table 1:** Population.

	Non-infected (NI)	Indeterminate (IND)	Cardiac (CARD)	Evolution (EV)
Total n	5	15	11	2
Male	2	8	7	1
Female	3	7	4	1
Evaluation interval, months (average)	36.6	63.2	63.2	63.5

### Ethics statement

The study was approved by the *Comitê de Ética em Pesquisa da Universidade Federal de Minas Gerais* (protocol COEP-ETHIC 502/11) and *Comitê de Ética em Pesquisas em Seres Humanos do Centro de Pesquisas René Rachou* (protocol CEPSH-CPqRR 15/2011). All enrolled individuals gave written informed consent prior to their inclusion in the study. All experiments were performed in accordance with relevant guidelines and regulations.

### Plasma samples

Peripheral blood samples were obtained at two distinct intervals between 1994 and 2014. All plasma was separated by centrifugation, aliquoted, and frozen at −80 °C until used.

### Multiplexed immunoassay

Plasma levels of TIMP-1, TIMP-2, TIMP-3, and TIMP-4, MMP-2, and MMP-9 were determined by Bio-Plex® Pro™ Human TIMP Assays and Bio-Plex® Pro™ Human MMP Assays (Bio-Rad, CA, USA), respectively, according to the protocol provided by the manufacturer.

### Statistical analysis

Statistical analysis was performed using GraphPad Prism 5.0 software package (San Diego, USA). All data files assume a non-Gaussian distribution and paired statistical comparisons were carried out using the nonparametric Wilcoxon signed-rank test and the Mann-Whitney test for comparison between T0 and T1. Low and high producers were separated using the median for T0 and T1 together. The percentage of high producers was determined considering the total number of frequencies (samples) of the times (T0 + T1) as 100%. The percentage was calculated by counting the number of high producers in each time. Biomarker sensitivity and specificity were determined by the Receiver Operating Characteristic curve (or ROC curve) between NI vs. IND, NI vs. CARD, and IND vs. CARD. In all cases, significance was considered at p < 0.05.
